# HIV-1 CRF01_AE strain is associated with faster HIV/AIDS progression in Jiangsu Province, China

**DOI:** 10.1038/s41598-017-01858-2

**Published:** 2017-05-08

**Authors:** Minjie Chu, Wuhong Zhang, Xuan Zhang, Wenjie Jiang, Xiping Huan, Xiaojun Meng, Bowen Zhu, Yue Yang, Yusha Tao, Tian Tian, Yihua Lu, Liying Jiang, Lei Zhang, Xun Zhuang

**Affiliations:** 1 Department of Epidemiology and Biostatistics, School of Public Health, Nantong University, Nantong, Jiangsu China; 2 School of Public Health and Preventive Medicine, Faculty of Medicine, Nursing and Health Sciences, Monash University, Melbourne, Australia; 3 Wuxi Municipal Centre for Disease Control and Prevention, Wuxi, Jiangsu China; 4 Department of Management Studies, University of Bath, Bath, UK; 5 Jiangsu Provincial Center for Disease Control and Prevention, Nanjing, Jiangsu China; 6 Melbourne Sexual Health Centre, Alfred Health, Melbourne, Australia; 7 Central Clinical School, Faculty of Medicine, Monash University, Melbourne, Australia; 8 Research Centre for Public Health, Tsinghua University, Beijing, China

## Abstract

The goal of this study was to assess risk factors associated with HIV/AIDS progression. Between May 2007 and December 2014, 114 subjects were enrolled in Wuxi City and examined every 6 months. The *pol* gene sequence was amplified to ascertain the HIV-1 subtype. A Cox proportional hazards regression model was used to estimate the factors associated with HIV/AIDS progression. The median follow-up time for all 114 subjects was 26.70 months (IQR: 18.50–41.47), while the median progression time of the 38 progressed subjects was 24.80 months (IQR: 14.13–34.38). Overall, the CRF01_AE subtype was correlated with a significant risk of accelerated progression compared to non-CRF01_AE subtypes (HR = 3.14, 95%CI: 1.39–7.08, *P* = 0.006). In addition, a lower CD4 count (350–499) at baseline was associated with a risk of accelerated HIV/AIDS progression compared to higher CD4 count (≥500) (HR = 4.38, 95%CI: 1.95–9.82, *P* < 0.001). Furthermore, interaction analyses showed that HIV-1 subtypes interacted multiplicatively with transmission routes or CD4 count at baseline to contribute to HIV/AIDS progression (*P* = 0.023 and *P* < 0.001, respectively). In conclusion, the CRF01_AE subtype and a lower CD4 count at baseline tend to be associated with the faster progression of HIV/AIDS. Understanding the factors affecting HIV/AIDS progression is crucial for developing personalized management and clinical counselling strategies.

## Introduction

Acquired immunodeficiency syndrome (AIDS) has been a major public health threat since its discovery in the United States in 1981. In general, the progression from human immunodeficiency virus (HIV) infection to AIDS development takes approximately 8–10 years^1^; however, this duration varies among individuals. Multiple factors have been found to contribute to the progression of HIV-1 infection, such as immunological, virological and host genetic factors^2–6^. In particular, the emergence of RNA sequencing technologies has provided a means for analysing the association between virological factors and HIV/AIDS progression.

The HIV-1 subtype has been associated with HIV/AIDS progression and has attracted much interest among researchers^7–11^. Unfortunately, no consensus has been reached in studies exploring the association between subtype and disease progression. Studies conducted in Tanzania and Uganda have suggested that subtype D is correlated with faster rates of CD4+ T-cell decline and disease progression than those observed in other subtypes and recombinant forms of the virus^7, 8^. However, a retrospective cohort study conducted during 1996 and 2007 revealed that Africans infected with B clade HIV-1 suffered from faster rates of HIV/AIDS progression compared with those infected with non-B clade subtypes^10^. Furthermore, a meta-analysis demonstrated that the trend of HIV/AIDS progression among different HIV-1 subtypes in a descending order was subtype C > D > AE > G > A^9^.

Though numerous prophylactic measures against HIV have been effectively employed in China^12^, they have not been able to efficiently terminate the pandemic. A systematic review and meta-analysis conducted in China showed that the pooled prevalence of CRF01_AE, subtype B, CRF07_BC, CRF08_BC, and subtype C was 44.54% (95% CI 40.81–48.30), 18.31% (95% CI 14.71–22.17), 16.45% (95% CI 13.82–19.25), 2.55% (95% CI 1.56–3.73), and 0.37% (95% CI 0.11–0.72), respectively^13^. Notably, the median time from the estimated date of seroconversion to the development of AIDS in the Chinese HIV-1 population was shorter compared with that in other countries^14–16^. Thus, understanding the factors that affect HIV/AIDS progression is vitally important for personalized disease management and clinical counselling. However, very few studies have explored the factors associated with HIV/AIDS progression among HIV-1 infected individuals in China. The aim of this study is to explore the natural history and molecular epidemiology of HIV-1 and to evaluate risk factors associated with HIV/AIDS progression.

## Results

### Demographic characteristics of subjects

**Figure 1:**
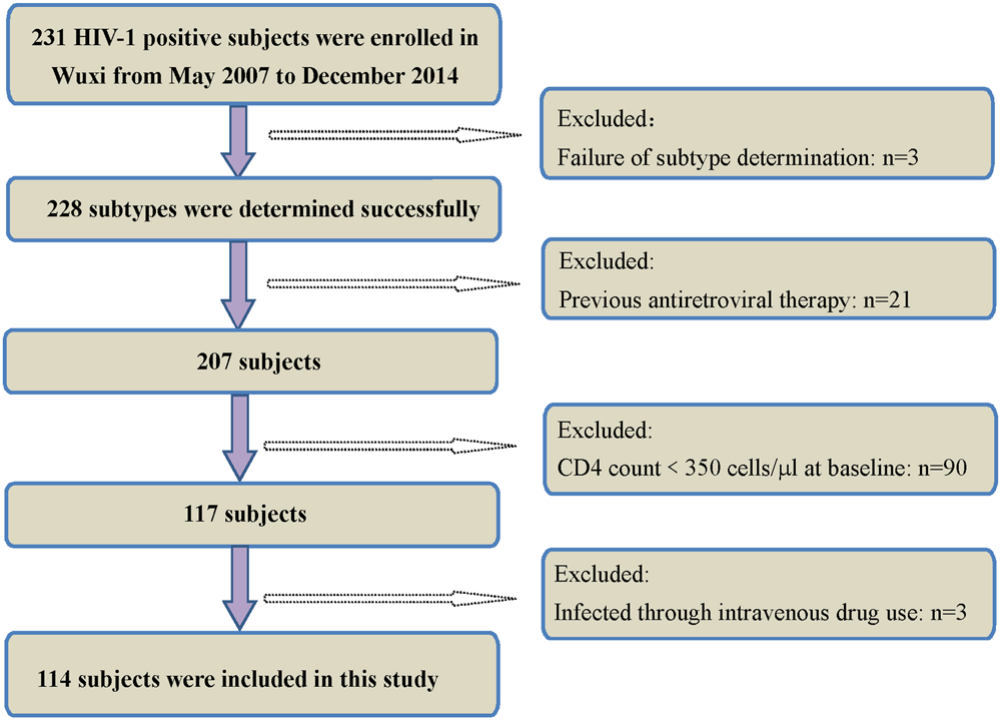
Flow chart of included and excluded study population.

**Figure 2:**
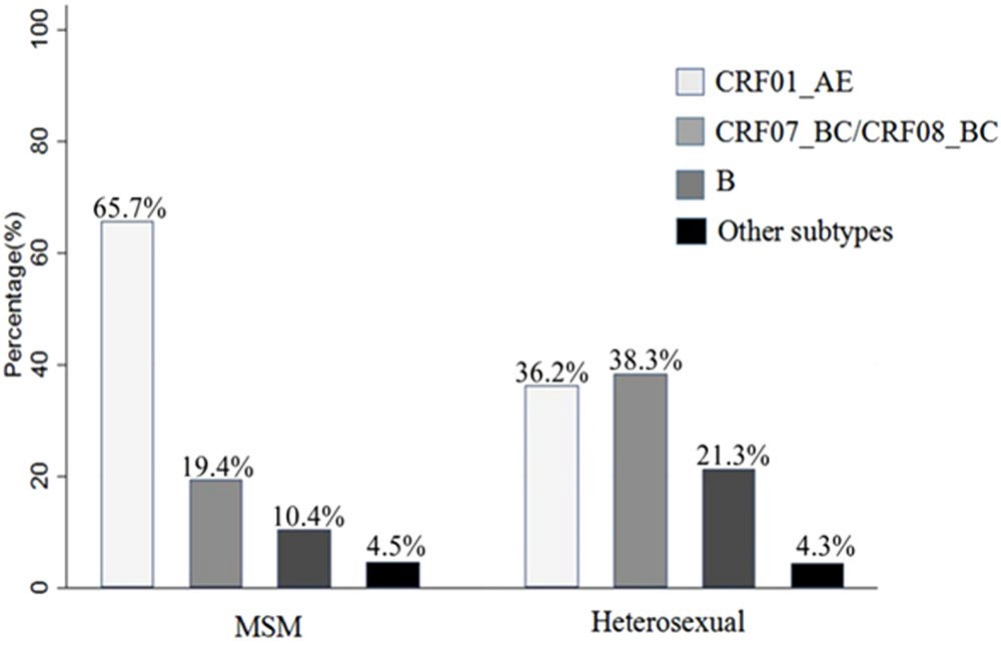
Distribution of HIV-1 subtypes in Wuxi.

Following the application of strict screening criteria, a total of 114 subjects were included in the analysis (Fig. 1). The majority of subjects were male (88.6%) and were of Han ethnicity (99.1%). The age of the subjects ranged from 15- to 74-years-old at the time of HIV-1 infection. The median CD4 count at baseline was 502.00 (IQR: 404.75–614.25). The median follow-up time was 26.70 months (IQR: 18.50–41.47), and the median progression time was 24.8 months (IQR: 14.13–34.38). The basic demographic data are summarized in Table 1. The distribution of HIV-1 subtypes in Wuxi was as follows: CRF01_AE (53.5%), B (14.9%), CRF07_BC/CRF08_BC (27.2%), and other subtypes (4.4%) (Fig. 2). Considering that the majority of the subjects were infected with CRF01_AE, we then separated the subjects into CRF01_AE and non-CRF01_AE groups. CRF01_AE was more prevalent in the MSM (men who have sex with men) group (65.7%, 44/67) than in the heterosexual group (36.2%, 17/47) (Fig. 2). There were significant differences between the subtypes with regard to gender, route of transmission, age at infection, and education status, all with *P* values less than 0.05 (Table 1).

**Table 1:** Demographic characteristics of 114 HIV-1 participants classified by viral subtypes in Wuxi.

Characteristics	All (n = 114)	CRF01_AE (n = 61)	Non-CRF01_AE (n = 53)	*P*
Gender (n, %)				**0**.**035**
Male	101 (88.6)	58 (95.1)	43 (81.1)	
Female	13 (11.4)	3 (4.9)	10 (18.9)	
Ethnicity (n, %)				0.465
Han	113 (99.1)	61 (100)	52 (98.1)	
Minority	1 (0.9)	0 (0)	1 (1.9)	
Route of transmission (n, %)				**0**.**002**
MSM	67 (58.8)	44 (72.1)	23 (43.4)	
Heterosexual	47 (41.2)	17 (27.9)	30 (56.6)	
Age at infection, yrs (n, %)				**0**.**007**
<30	59 (51.8)	35 (57.4)	24 (45.3)	
30-	19 (16.7)	14 (22.9)	5 (9.4)	
≥40	36 (31.6)	12 (19.7)	24 (45.3)	
Marriage Status (n, %)				0.300
Single	52 (45.6)	31 (50.8)	21 (39.6)	
Married	37 (32.5)	16 (26.2)	21 (39.6)	
Divorced/widowed	25 (21.9)	14 (23.0)	11 (20.8)	
Education (n, %)				**0**.**014**
Primary school or below	12 (10.5)	2 (3.3)	10 (18.9)	
Junior/high school	65 (57.0)	35 (57.4)	30 (56.6)	
College or above	37 (32.5)	24 (39.3)	13 (24.5)	
CD4 count at baseline (n, %)				0.260
≥500	59 (51.8)	35 (57.4)	24 (45.3)	
350–499	55 (48.2)	26 (42.6)	29 (54.7)

### Factors associated with HIV/AIDS progression

In the Cox proportional hazards regression model, the CRF01_AE subtype was correlated with a significantly accelerated rate of HIV/AIDS progression compared to non-CRF01_AE subtypes (HR = 3.14, 95%CI: 1.39–7.08, *P* = 0.006) (Table 2). In addition, a lower CD4 count (350–499) at baseline was also significantly associated with HIV/AIDS progression compared to a higher CD4 count (≥500) (HR = 4.38, 95% CI: 1.95–9.82, *P* < 0.001) (Table 2).

**Table 2:** Factors associated with HIV/AIDS progression (defined as time for CD4+ T-cell count decrease to <350 cells/μL) using Cox proportional hazards model.

Covariates	Progressor/Total	Median progression time (Months)	HR (95%CI)	*P*ª
Gender				
Male	31/101	25.33	1	
Female	7/13	9.13	1.48 (0.51–4.31)	0.475
Route of transmission				
MSM	18/67	25.26	1	
Heterosexual	20/47	24.25	1.33 (0.60–2.97)	0.486
Age at infection, yrs				
<30	15/59	26.93	1	
30-	5/19	16.53	1.41 (0.50–3.99)	0.519
≥40	18/36	24.78	1.76 (0.75–4.11)	0.191
Viral subtype				
Non-CRF01_AE	16/53	26.13	1	
CRF01_AE	22/61	22.15	3.14 (1.39–7.08)	**0**.**006**
Education				
Primary school or below	7/12	24.13	1	
Junior/high school	24/65	27.41	3.24 (0.92–11.36)	0.067
College or above	7/37	19.93	1.72 (0.36–8.09)	0.495
CD4 count at baseline				
≥500	8/59	26.06	1	
350–499	30/55	24.26	4.38 (1.95–9.82)	**<0**.**001**

Abbreviations: HR, hazard ratio; CI, confidence intervals. ^ª^Cox regression with adjustment for gender, route of transmission, age at infection, viral subtype, education and CD4 count at baseline where necessary.

**Figure 3:**
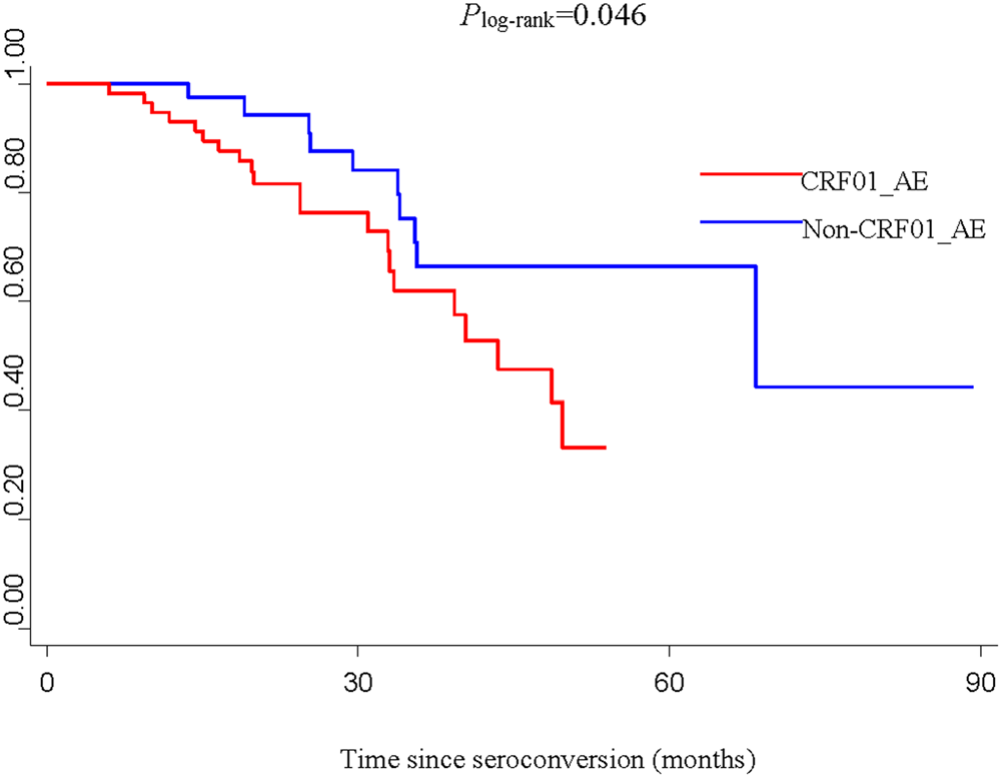
Kaplan-Meier survival curves for progression from estimated date of seroconversion to CD4 count <350 cells/μL in different subtypes of male group.

**Figure 4:**
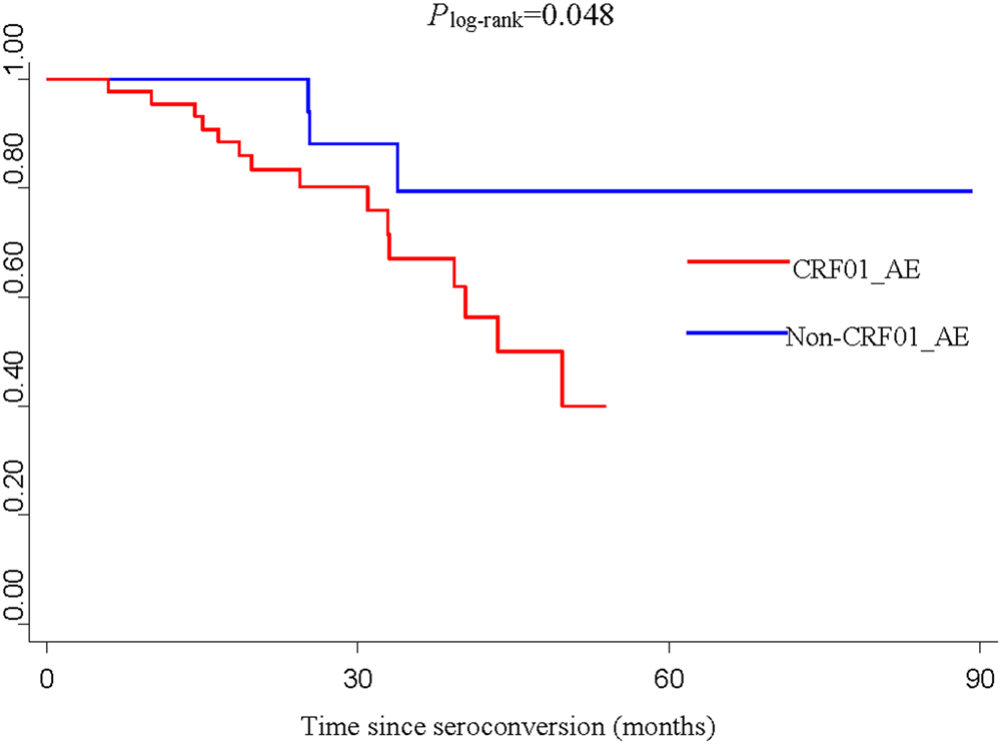
Kaplan-Meier survival curves for progression from estimated date of seroconversion to CD4 count <350 cells/μL in different subtypes of MSM group.

**Figure 5:**
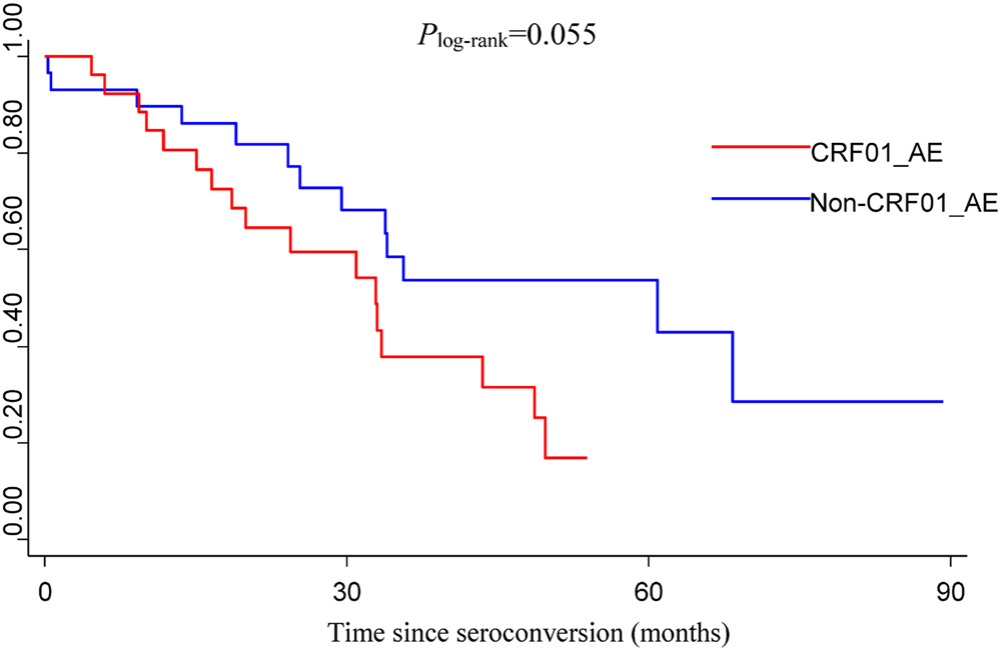
Kaplan-Meier survival curves for progression from estimated date of seroconversion to CD4 count <350 cells/μL in different subtypes of lower baseline CD4 count group (350–499).

Considering the significant differences in gender, route of transmission, age at infection and education between the CRF01_AE and non-CRF01_AE groups (*P* < 0.05), stratification analyses were performed to explore the association between the 4 factors above and HIV/AIDS progression. We found that HIV/AIDS progressed faster in male subjects infected with CRF01_AE compared to males infected with non-CRF01_AE subtypes (*P* = 0.046) (Fig. 3). In addition, MSM patients infected with CRF01_AE exhibited a significantly faster progression rate than MSM patients infected with non-CRF01_AE subtypes (*P* = 0.048) (Fig. 4), while no significant difference between HIV/AIDS progression in CRF01_AE and non-CRF01_AE subtypes was observed in heterosexuals (*P* = 0.405). Further, no significant association was found between HIV-1 subtype and progression in any of the age groups, nor for either of the education groups. In addition, since lower CD4 count at baseline was a risk factor for fast HIV/AIDS progression, in concordance with the well-known knowledge, we also performed the stratified analysis of CD4 count at baseline (divided into two groups: ≥500 and 350–499) to explore the association between HIV subtypes and progression and found that HIV/AIDS progressed borderline significantly faster in subjects with lower baseline CD4 count (350–499) infected with CRF01_AE compared to those with lower baseline CD4 count (350–499) infected with non-CRF01_AE subtypes (*P* = 0.055) (Fig. 5), however, no significant association was found between HIV-1 subtype and progression in the higher baseline CD4 count group (≥500).

Considering the significant difference in the route of disease transmission between the CRF01_AE and non-CRF01_AE groups, we investigated whether the effect of HIV-1 subtype on HIV/AIDS progression was modified by transmission route. Interaction analyses showed that HIV-1 subtypes interacted multiplicatively with transmission route to contribute to HIV/AIDS progression (interaction *P* = 0.023) (Table 3). In addition, since lower CD4 count at baseline was a risk factor for fast HIV/AIDS progression, in concordance with the well-known knowledge, we also investigated the interaction of HIV-1 subtypes and CD4 count at baseline. As expected, interaction analyses showed that HIV-1 subtypes interacted multiplicatively with CD4 count at baseline to contribute to HIV/AIDS progression (interaction *P* < 0.001) (Table 4).

**Table 3:** The interaction between HIV-1 subtypes and transmission route on HIV/AIDS progression.

Subtype	Transmission route	Progressor/Total	Median progression time (Months)	HR (95%CI)	*P* ^a^
Non-CRF01_AE	MSM	3/23	25.33	1	
Non-CRF01_AE	Heterosexual	13/30	26.93	1.72 (0.41–7.27)	0.463
CRF01_AE	MSM	15/44	24.40	3.19 (0.89–11.36)	0.074
CRF01_AE	Heterosexual	7/17	19.93	4.52 (1.11–18.47)	0.036
*P* for multiplicative interaction					0.023

^a^ *P* value of interaction analysis between HIV-1 subtype and transmission route on HIV/AIDS progression with adjustment for gender, age at infection and baseline CD4 count.

**Table 4:** The interaction between HIV-1 subtypes and CD4 count at baseline on HIV/AIDS progression.

Subtype	CD4 count at baseline	Progressor/Total	Median progression time (Months)	HR (95%CI)	*P* ^a^
Non-CRF01_AE	≥500	3/24	26.93	1	
Non-CRF01_AE	350–499	13/29	25.33	3.18 (0.90–11.28)	0.073
CRF01_AE	≥500	5/35	24.37	2.09 (0.47–9.27)	0.331
CRF01_AE	350–499	17/26	19.93	10.99 (2.95–41.01)	<0.001
*P* for multiplicative interaction					<0.001

^a^ *P* value of interaction analysis between HIV-1 subtype and CD4 count at baseline on HIV/AIDS progression with adjustment for gender, age at infection and transmission route.

## Discussion

In the present study, in order to understand the natural history and disease progression of HIV-1, we studied HIV/AIDS progression in subjects infected with different subtypes of HIV-1 in Wuxi, Jiangsu by measuring the length of time between the estimated date of seroconversion to the time of a decrease in CD4 count to <350 cells/μL. The subtype distribution in our study was 53.5% CRF01_AE, 14.9% B, 27.2% CRF07_BC/CRF08_BC and 4.4% other subtypes, which was consistent with previous reports^17, 18^, indicating that recombinant forms of the virus might be on the rise worldwide, which have already been associated with faster disease progression as seen in Cuba^19^ and Brazil^20^. Most importantly, our study demonstrated that CRF01_AE is associated with faster HIV/AIDS progression, which is in agreement with a previous study conducted in China^14^. In another study, Ng *et al*. (2011) showed that CRF01_AE-infected seroconverters experienced a faster rate of CD4+ T-cell decline and thus required earlier cART initiation compared to non-CRF01_AE patients^21^. It remains unclear as to why CRF01_AE was associated with rapid CD4 decline. However, some studies have shown that a high proportion of X4 tropism is observed in the CRF01_AE subtype, and numerous studies have demonstrated that X4 tropism is associated with increased rates of CD4 count decline and progression to advanced immunosuppression^22–24^. In addition, it has been postulated that the decline of the host immune system after HIV-1 infection may allow X4 viruses to evolve and replicate freely^25^, which may explain the increased rate of disease progression in CRF01_AE patients.

In our study, we found that a lower CD4 count (350–499 cells/μL) at baseline was a risk factor for fast HIV/AIDS progression, in concordance with previous studies^26^. CD4+ T cells are crucial in forming an immune response to foreign antigens, and they are the primary target cells of the HIV-1 virus. The continuous loss of CD4+ T cells could eventually result in the loss of ability to mount an effective immune response to pathogens, as well as the deaths of subjects in the terminal stage of HIV infection^27^.

The impact of transmission route on disease progression has long been a subject of debate^28^. In our study, no significant relationship between transmission route and HIV-1 progression was observed (*P* = 0.486); this finding is inconsistent with a previous study conducted in China by Li *et al*., which revealed that the transmission route in MSM subjects is an independent risk factor for the progression of HIV infection to AIDS^14^. There are several possible reasons for the difference in our results. First, differences in the eligibility criteria of the studied populations may have affected the outcome; we included subjects with CD4 > 350 cells/μL at baseline and defined HIV/AIDS progression as CD4 < 350 cells/μL, whereas the inclusion criteria used by Li *et al*. was a CD4 count <350 cells/μL and they defined HIV/AIDS progression as CD4 < 200 cells/μL. Second, the difference may have resulted from the relatively short follow-up time in our study.

To the best of our knowledge, although the data are not new, this is the first study of the natural course of HIV-1 infection in antiretroviral-naive subjects in Jiangsu, China. Interestingly, this is the first report of transmission routes interacting multiplicatively with HIV-1 subtypes to contribute to HIV/AIDS progression. Moreover, our study may have important implications for personalized disease management and clinical counselling in other Asian countries since the CRF01_AE strain is dominant in Asia. However, our study has limitations that need to be addressed. First, we defined CD4 < 350 cells/μL as a time-to-event endpoint in HIV/AIDS progression, and we could not exclude the possibility that the standards of baseline CD4 count and progression CD4 count were so approach that the judgment of HIV/AIDS progression might be not specific enough. Further study measuring viral load and the clinical symptoms of AIDS should be conducted. Second, in the current sample size (114 subjects), the statistical power is approximately 49.8% in detecting an effect size of 1.50 with an α-level of 0.05 for the association between subtype and HIV/AIDS progression. Nevertheless, we could not exclude the possibility that a small sample size may result in low statistical power. Therefore, further studies with larger sample sizes are needed to confirm our findings. Third, the differing rates of HIV/AIDS progression in HIV-infected individuals are complex and involve various factors, with viral and host factors being the most impactful; thus, further studies are needed to extensively explore additional factors (e.g., viral load and host genetics)^8, 15, 29, 30^. Fourth, we inferred HIV-1 subtype by sequencing the *pol* region, which could result in the misclassification of recombinants as pure subtypes. It is also possible that some of the reported recombinants are actually HIV-1 dual/multiple infections, which have been associated with faster disease progression^31, 32^. In addition, we only sequenced the *pol* region and have not sequenced *env* region at present, thus no data are available about the co-receptor use by HIV-1. Therefore, whole genome sequencing is the ideal method for inferring subtype and performing co-receptor use analysis. Finally, we observed that CRF01_AE is associated with faster HIV/AIDS progression. However, we were unable to elucidate the biological mechanisms underlying this phenomenon; thus, further functional evaluations are necessary.

## Conclusions

This study revealed that subjects carrying the CRF01_AE subtype may suffer a faster loss of CD4 cells and earlier HIV/AIDS progression compared with those carrying a non-CRF01_AE subtype. Regular surveillance of HIV-1 subtypes and CD4 count would be beneficial in monitoring HIV/AIDS progression, as well as for improving personalized disease management and clinical counselling. Further studies incorporating subjects with different ethnic backgrounds combined with functional evaluations are warranted to verify the relationship between HIV-1 subtypes and HIV/AIDS progression.

## Methods

### Ethics Statement

This study was reviewed and approved by the Institutional Review Board at the Human Medical Research Ethics Committee of the Center for Disease Control and Prevention (CDC) in Wuxi, and written informed consent was obtained from every subject. The methods were carried out in accordance with the approved guidelines.

### Study population

A total of 231 subjects were enrolled in the study between May 2007 and December 2014 in Wuxi with follow-up intervals of 6 months. CD4 count was determined at each visit. Follow-up started in May 2007, and the last follow-up was concluded in December 2014. Subjects were included if they met the following eligibility criteria: (1) CD4 count was higher than 350 cells/μL at baseline; (2) subject was antiretroviral-naive; (3) HIV was contracted through sexual infection; and (4) the subjects attended subsequent follow-ups from the estimated date of seroconversion (Fig. 1). HIV/AIDS progression was defined as a CD4 count less than 350 cells/μL. Ultimately, a total of 114 individuals were included in this study. Clinical data, including basic demographic information and CD4 counts, were collected from medical charts at the Wuxi CDC. Basic demographic information of the subjects included age at HIV-1 infection, gender, ethnicity, marital status, education status and route of HIV-1 transmission.

### Viral RNA extraction, PCR amplification and sequencing

All blood samples were processed at the Shanghai Municipal CDC for population-based nucleotide sequencing of plasma HIV-1 RNA encoding regions of reverse transcriptase (RT) and protease (PR) genes. HIV-1 RNA was extracted from 200 µl of stored plasma specimen using the QIAmp Viral RNA Mini kit (Qiagen, Valencia, CA, USA) per the manufacturer’s instructions. RT-PCR and nested polymerase chain (nPCR) amplification for the *pol* region were performed according to previously published methods^17, 33^. Subtypes were determined using the Recombinant Identification Program (http://www.hiv.lanl.gov/content/sequence/RIP/RIP.html).

### Statistical analysis

Baseline characteristics were analysed by using the χ^2^ test for categorical variables. The Cox proportional hazards regression model was used to estimate risk factors associated with the time of CD4 count decrease to <350 cells/μL. *P* < 0.05 was considered statistically significant. The SPSS 17.0 statistical package and STATA 13 were used for all analyses.
